# Präsenzlehre an Universitäten und Hochschulen unter den Bedingungen der SARS-CoV-2-Pandemie: Empfehlungen zu technischen, organisatorischen und persönlichen Schutzmaßnahmen im Rahmen des Arbeitsschutzes

**DOI:** 10.1007/s40664-020-00411-9

**Published:** 2020-10-29

**Authors:** Alexandra M. Preisser, Julia Pieter, Volker Harth

**Affiliations:** Zentralinstitut für Arbeitsmedizin und Maritime Medizin (ZfAM), Universitätsklinikum Hamburg-Eppendorf, Seewartenstraße 10, 20459 Hamburg, Deutschland

**Keywords:** Infektionsgefährdung, Arbeitsschutz, COVID-19, Studium, Unterricht, Risk of transmission, Occupational safety, COVID-19, Study, Teaching

## Abstract

Zur Aufrechterhaltung des universitären Lehrangebotes unter Einhaltung der Abstandsregeln entsprechend des Arbeitsschutzstandards des Bundesministeriums für Arbeit und Soziales (BMAS) sind unter den Bedingungen der COVID-19-Pandemie beim Aufeinandertreffen von Studierenden und Lehrkräften in großen Gruppen (Hörsaal) und kleinen Lehrräumen (Seminare, Praktika) besondere Regelungen und Maßnahmen zu beachten. Der Artikel gibt detaillierte Empfehlungen für die praktische Umsetzung von Schutz- und Präventionsmaßnahmen nach dem im Arbeitsschutz üblichen STOP-Prinzip: Substitution, Technische, Organisatorische und Persönliche Schutzmaßnahmen. Hierbei werden die Verantwortlichkeiten benannt und auch schwierige Situationen, in denen körperliche Nähe notwendig ist (z.B. Lehre medizinischer Untersuchungen, gemeinsame Laborarbeit, Sport- und Tanzseminare), hinsichtlich der notwendigen Maßnahmen analysiert und Empfehlungen formuliert.

## Abstract (en)

In order to maintain university teaching while observing the distance rules in accordance with the occupational health and safety standard of the German Federal Ministry of Labour and Social Affairs (BMAS), special regulations and measures must be observed under the conditions of the COVID-19 pandemic when students and teaching staff meet in large groups (lecture hall) and small teaching rooms (seminars, internships). The article gives detailed recommendations for the practical implementation of protective and preventive measures according to the STOP principle, which is customary in occupational health and safety: substitution, technical, organisational and personal protective measures. Responsibilities are named and also difficult situations where physical proximity is necessary (e.g. teaching medical examinations, joint laboratory work, sports and dance seminars) are analysed with regard to the necessary measures; recommendations are formulated.

## Kernbotschaften

Um Präsenzlehre unter Covid-19-Pandemiebedingungen sicher durchzuführen ist eine Gefährdungsbeurteilung verschiedener Settings im Lehrbetrieb notwendig. Die Empfehlungen sind derzeit nur analog zu bisherigen Erfahrungen im Arbeits- und Gesundheitsschutz zu formulieren:

- Die Gewährleistung des Sicherheitsabstandes von 1,5 m zwischen Lehrenden und Studierenden sowie den Studierenden untereinander kann bei den meisten Lehrformen sichergestellt werden. Unterrichts- und Aufenthaltsräume müssen eine entsprechende Größe aufweisen, die Lehrgruppen ggf. verkleinert werden.
- Es wird empfohlen, Mund-Nasen-Bedeckung (MNB) bei Zugang und Verlassen der Unterrichtsräume sowie in Aufenthalts- und Toilettenräumen grundsätzlich zu etablieren.
- Im Falle von nahem oder unvermeidbarem Körperkontakt sind die Teams klein zu halten, es muss Mund-Nasen-Bedeckung getragen werden und die Personen sollten nicht zwischen verschiedenen Teams wechseln.
- Besonders schwer umsetzbar sind Unterrichtsformen mit Patient*innen-Kontakt und anderen Personen, welche als Risikopersonen angesehen werden müssen. Mindeststandard muss hier Schutzkleidung mit Kittel und medizinischer Mund-Nasen-Schutz (MNS) für Studierende und Patient*innen sein.

Der Artikel richtet sich v. a. an Entscheidungsträger*innen und die für Arbeitsschutz und Hygiene verantwortlichen Personen in der Lehre an Universitäten und Hochschulen.

Die Empfehlung ist in weiten Teilen auch auf andere Formen der Erwachsenenbildung zu übertragen.

## Hintergrund

Im Rahmen der nationalen Maßnahmen zur Verhinderung einer schnellen Ausbreitung der Infektion mit SARS-CoV‑2 haben die Länder ihre Universitäten und Hochschulen angewiesen, den Lehrbetrieb zu reduzieren und Online-Lehre anzubieten. Entsprechend des *Einheitlichen Arbeitsschutzes gegen das Corona-Virus* des Bundesministeriums für Arbeit und Soziales (BMAS) [1] sollen durch passende Maßnahmen des Arbeits- und Infektionsschutzes größere Menschenansammlungen vermieden und die Einhaltung der Abstandsregeln sichergestellt werden. Neben den klassischen Vorlesungen bietet die moderne Lehre an Hochschulen und Universitäten viele weitere Formate: Seminare, studentische Diskussionsgruppen, strukturierte Praktika in Versuchslaboren (MINT-Fächer^1^), Kleingruppenunterricht (im Betrieb, Krankenhaus), Selbststudium in Bibliotheken und E‑Learning. Die Einhaltung der Abstandsregeln entsprechend des Arbeitsschutzstandards des BMAS [1] ist v. a. beim Aufeinandertreffen von Studierenden und Lehrkräften in großen Gruppen (Hörsaal) und kleinen Lehrräumen (Seminare, Praktika) zu ermöglichen. Besonders herausfordernd ist die Einhaltung des Infektionsschutzes bei sehr engem Abstand, evtl. sogar Körperkontakt untereinander (Schauspiel und Tanz, medizinische Kurse), oder mit Proband*innen und Patient*innen, die nicht Beschäftigte der Hochschule sind.

Die Empfehlungen der nationalen Akademie der Wissenschaften Leopoldina in der dritten Ad-hoc-Stellungnahme vom 13.04.2020 lauteten: „An den Universitäten und Hochschulen sollte das Sommersemester weitgehend als Online/Home-learning-Semester zu Ende geführt werden. Fließende Übergänge und Mischungen von Fern- und Präsenzunterricht bieten sich an. Voraussetzung sind abgestimmte Lerneinheiten, die digital vermittelt werden“ [2].

Anders als in anderen Bildungseinrichtungen, wie z. B. Grundschulen, ist den erwachsenen Studierenden zu unterstellen, dass sie in der Rolle als Beschäftigte der Universität mündig und verantwortungsbewusst für sich und zum Schutz der anderen Studierenden und Beschäftigten handeln können, außerdem bereit sind, auch alternative Lernformen auszuprobieren und weiterzuentwickeln. Demgegenüber stehen Lehrkräfte, die sich ggf. ebenfalls in die Nutzung digitaler Lehrmedien einarbeiten müssen. Auch könnten diese – in Einzelfällen auch Studierende – der Risikogruppe für einen schweren Verlauf von COVID-19 angehören. In der aktuellen Pandemie müssen daher die Hochschulen die Anforderungen in den Lernzielkatalogen und die Vorgaben für das Erreichen des Berufsabschlusses neu betrachten und ggf. Ausnahmeregelungen treffen.

## Ziel der Stellungnahme

Der Artikel hat das Ziel, Wissen zusammenzutragen, wie präventiv die Tätigkeiten in der universitären Lehre für die Lehrenden, die Studierenden und weitere beteiligte Personen möglichst sicher sowie physisch und psychisch wenig belastend im Hinblick auf das COVID-19-Infektionsrisiko gestaltet werden können. Es richtet sich an die Verantwortlichen des Lehrbetriebes; sie haben Arbeits- und Infektionsschutzmaßnahmen zur Minderung des Risikos einer SARS-CoV-2-Infektion innerhalb des universitären Betriebes anzuordnen. Für besonders vulnerable Personengruppen soll nicht nur eine Minderung, sondern eine Minimierung des Infektionsrisikos durch die vorgeschlagenen Maßnahmen erreicht werden. Der Artikel soll dazu beitragen, die erfolgreiche Fortführung und den Abschluss des Studiums unter den neuen Voraussetzungen zu ermöglichen.

## Methoden und Lösungsansatz

Die Autor*innen haben anhand einer internetbasierten Suche nach aktuellen Empfehlungen und Erkenntnissen zu dem Thema und benachbarter Themen durchgeführt, diese zusammengeführt und durch eigene, erfahrungsgestützte Detailüberlegungen zu spezifischen Lehrformen ergänzt. Die gefundene Anzahl relevanter Internet-basierter Informationen und Literatur zu diesem Thema wächst aktuell stetig, weswegen die Autor*innen keinen Anspruch auf Vollständigkeit erheben können. Als Lösungsansatz werden die bereits Internet-basiert veröffentlichten und verordneten Hygienemaßnahmen zur COVID-19-Pandemie mit den Arbeitsschutzmaßnahmen entsprechend des STOP-Prinzips kombiniert. Vorliegende Empfehlungen beruhen auf Expert*innenmeinung sowie Vorgaben der entsprechenden Behörden. Es gibt zum derzeitigen Zeitpunkt keine ausreichende quantitative Evidenz für die Wirksamkeit dieser Maßnahmen im Kontext von Infektionen mit SARS-CoV‑2.

## Hygienemaßnahmen

Ziel der Hygienemaßnahmen ist der Schutz am Arbeitsplatz bzw. im Studium vor der Infektion mit SARS-CoV‑2. Daher ist es wichtig, dass jede Person, die dem Bildungsbetrieb an Universitäten und Hochschulen als Beschäftigte/r bzw. Studierende/r angehört, diese allgemeinen Verhaltensregeln kennt. Die Bildungseinrichtungen sollten regelmäßig hierüber informieren und unterweisen (bspw. in Einführungsvorlesungen). Die Hochschulen und Universitäten müssen für die Umsetzung der Kernelemente in allen Bereichen der Hochschulen und Universitäten Sorge tragen. Umfangreiche Informationen stellen sowohl das Robert-Koch-Institut [3, 4] als auch die Bundeszentrale für gesundheitliche Aufklärung [5] zur Verfügung.

Folgende Inhalte sollten die Unterweisungen enthalten:

- Direkten Kontakt meiden
  - Einhaltung von mindestens 1,5 m Abstand zu anderen Personen.
  - Vermeiden der Berührung von Augen, Nase oder Mund mit ungewaschenen Händen.
  - Vermeiden von Begrüßungen durch Umarmungen, Küssen und Händeschütteln.
  - Vergrößern des Sprechabstandes.
- Richtiges Händewaschen
  - Waschen der Hände häufiger, besonders vor dem Zubereiten und Verzehr von Speisen, nach dem Toilettengang oder dem nach Hause kommen. Auch nach dem Erreichen des Veranstaltungsortes (zum Beginn von Seminaren nach Raumwechsel oder nach Pausen) ist die Reinigung der Hände zu empfehlen. Genügend Waschräume sollten zur Verfügung stehen. Auch in diesen ist die Einhaltung der Abstandsregeln, z. B. durch Reduzierung und Vorgaben zur maximalen Personenzahl in den Wasch- und Toilettenräumen zu empfehlen.
  - Die Hände unter fließendes Wasser halten, Seife 20 bis 30 s auch zwischen den Fingern verreiben, dann sorgfältig abspülen und abtrocknen mit Einmaltrockentüchern.
- Hygienisches Husten und Niesen
  - Nach dem Husten und Niesen keine Berührung von Gegenständen oder Mitmenschen, Hände waschen.
  - Beim Husten oder Niesen größtmöglichen Abstand zu anderen Personen einhalten.
  - Husten in die Armbeuge oder Verwendung eines Taschentuches, dieses nur einmal benutzen und sofort entsorgen.

## STOP-Prinzip als Leitfaden zur Umstrukturierung

Das im Arbeitsschutz verankerte STOP^2^-Prinzip ist eine Möglichkeit, die verschiedenen Bereiche der Hochschullehre entsprechend der Gefährdungsbeurteilung strukturiert zu betrachten; es kann in der Gestaltung einzelner Bereiche helfen, um eine Minderung des SARS-CoV-2-Infektionsrisikos der Beschäftigten und Studierenden zu erreichen. Das Prinzip gibt die Rangfolge der Schutzmaßnahmen wieder: (1) den Ersatz (Substitution, z. B. durch Online-Angebote) oder den Wegfall nichtdringlicher Aufgaben und Tätigkeiten, (2) die Umsetzung technischer und organisatorischer Maßnahmen zur Einhaltung der Abstandsregeln und außerdem, sollten Letztere aufgrund unvermeidbarer enger Personenkontakte nicht möglich sein, (3) weitere Regelungen zu persönlichen Schutzmaßnahmen.

Die generellen Prinzipien des Infektionsschutzes vor SARS-CoV-2-Infektionen, nämlich

- Kontakte reduzieren und Abstandsregeln einhalten im Unterricht und im Kontakt mit weiteren Beteiligten,
- Reduzierung von Miterkrankungen oder Quarantänefällen durch kleine und feste Teams und
- Tragen persönlicher Schutzkleidung, wenn erforderlich,

können mit dem STOP-Prinzip folgendermaßen umgesetzt werden:

### Substitution

Substituierende Maßnahmen können mögliche infektiöse Kontakte mit anderen Studierenden oder Beschäftigten am effizientesten reduzieren. Dies erfordert die Überprüfung und Entscheidung durch die Leitungsverantwortlichen der Hochschule, ob bisherige Arbeitsweisen durch andere Lehr- und Kommunikationsformen ersetzt werden können. Entsprechende Anordnungen können auch kurzfristig angepasst werden. Maßnahmen der Substitution sind beispielsweise:

- Lernzielkataloge und Lehrangebote überprüfen: Priorisierung der Lehrangebote, die für das weitere Studium und die Abschlüsse essenziell sind.
- Vorlesungen und Seminare durch digitale Angebote ersetzen (Präsentationen mit Tonspur, Webinare, online-Lehrmaterialien für das Selbststudium).
- Bereitstellung von Videokonferenzprogrammen für studentische Diskussionsrunden (mit Schulungen).
- Überprüfen, ob auch Praktika durch digitale Angebote ersetzt werden können (online-Diskussionsrunden; Demonstrationen über Videos, Bereitstellung von geeigneten Lehrmaterialien in Printform, in digitalen Medien der Bibliotheken oder Angabe geeigneter Internetangebote).
- Präsenzpflichten auf Notwendigkeit überprüfen. Teamsitzungen und Besprechungen per Video- oder Telefonkonferenz.
- Die im Ablauf des Studiums notwendigen Antragstellungen online ermöglichen, anstelle des persönlichen Erscheinens vor Ort.
- Telefonische Beratungen anbieten.

### Technische Anpassungen

- Einrichtung der technischen Voraussetzungen für die Durchführung von Home Teaching and Learning (Software Lizenzen).
- Anpassung der räumlichen Gegebenheiten: z. B.
  - Einbau von Trennwänden, Abstandshaltern, Plexiglastrennscheiben für Praktikumsräume,
  - Gewährleistung einer ausreichenden Belüftung,
  - Zugang zu Händewasch-Möglichkeiten, ausreichend Seife und Papier,
  - Durchgangstüren, soweit möglich, automatisch öffnend/offenlassen,
  - personenbezogene Arbeitsplätze, Benutzung von Tastatur, Maus, Arbeitsmaterialien etc. durch mehrere Personen möglichst vermeiden,
  - Abstände zwischen Sitzmöglichkeiten in Unterrichtsräumen, Aufenthaltsräumen, Bibliotheken etc. überprüfen (mindestens 1,5–2 m). Aushang der maximalen Personenzahl an der Eingangstür des Unterrichtsraumes.
- Anpassung der Reinigungspläne (Hinweise zu Reinigung und Desinfektion; [3]):
  - Intensivierte, häufige Reinigung von Kontaktflächen (Türklinken).

### Organisatorische Anpassungen

**Figure 1:**
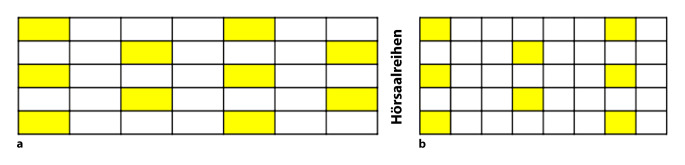

- Wiederholte Information und Aufklärung der Mitarbeitenden und Studierenden über die vorgesehenen und einzuhaltenden Maßnahmen zum Infektionsschutz sowie zum Verhalten im Krankheitsfall bzw. bei ersten möglichen Krankheitszeichen (Husten, Halsschmerzen, Fieber, Geschmacksverlust), z. B. durch Aushänge an den Eingängen der Gebäude und in den Toiletten/am Waschbecken bzgl. richtigem Händewaschen, durch Online-Angebote oder Einführungsfolien bei Seminaren etc.
- Reduktion der Gruppengrößen entsprechend der Größe und Möblierung der Unterrichtsräume zur Einhaltung des notwendigen Abstandes von 1,5–2 m.
- Reduktion der Kontaktzeiten zwischen den Mitarbeitenden und den Studierenden bei Raumwechsel z. B. durch zeitliche Entzerrung der Stundenpläne, exakte Absprachen bezüglich Nutzungszeiten von Räumlichkeiten.
- Vorgabe der Wechselwege (z. B. nur von einer Seite in die Reihe eintreten, zur anderen Seite aus der Reihe austreten), Kennzeichnung mit Richtungspfeilen in den Aufgängen und Abgängen, Trennung der Verkehrswege durch Bodenmarkierungen.
- Kennzeichnung der zu nutzenden Sitzplätze in den Hörsälen, jede Reihe nutzen (ca. jeder 4. Bis 6. Platz je nach Sitzbreite, versetzt zur vorherigen Reihe, somit Abstandswahrung nach schräg vorne bzw. hinten von je 1,5–2 m; Abb. 1); auch, damit im Falle eines Austretens möglichst wenige weitere Studierende mit aufstehen und austreten müssen.
- Gewährung von Home Learning & Teaching, wenn möglich.
- Einteilen von festen Studierendengruppen und Betreuungspersonen, damit im Erkrankungsfall nur eine begrenzte Zahl an Kontaktpersonen in Frage kommt und die Infektionsermittlung effektiv durchgeführt werden kann.
- Festlegung des weiteren Vorgehens (Dienstanweisung o. Ä.) bei Auftreten eines COVID-19-Erkrankungsfalls in der Hochschuleinrichtung mit Benennung fester Ansprechpartner*innen für Hygienefragen sowie für die Organisation und Umsetzung der Maßnahmen. Zur Vorbereitung hierauf gehört auch das Führen von Teilnehmerlisten und ggf. Erfassung der privaten Telefonnummern (oder Sicherstellung einer Einsicht in die Kontaktdaten durch den Krisenmanager auch am Wochenende oder abends außerhalb von Sekretariatsöffnungszeiten), sodass betroffene Personen schnell informiert werden können. Teilnehmerlisten sind datenschutzkonform zu erstellen und aufzubewahren. Ein entsprechendes Konzept ist erforderlich.
- Einhalten der räumlichen Trennung der einzelnen Teams auch während der Pausenzeiten.

### Persönliche Verhaltensregeln

- Einhalten der Abstandsregeln, auch während der Pausenzeiten.
- Husten- und Niesetikette (in Ellenbeuge) ist einzuhalten.
- Mitarbeitende und Studierende mit Symptomen einer akuten Atemwegserkrankung sollen der Universität oder der Hochschule fernbleiben.

## Besondere Lehrformen und Arbeitsräume

### Praktika (Labore, technische Räume)

In diesen Lehrveranstaltungen sind im Wesentlichen die Abstandsregeln einzuhalten, wie oben beschrieben. Dies erfordert kleine Gruppen; die ursprünglichen Gruppengrößen müssen entsprechend aufgeteilt und die Veranstaltungshäufigkeit entsprechend erhöht werden. Die begrenzten Kapazitäten von Räumen und Dozierenden können folgend dazu zwingen, dass andere Praktika aktuell weiterhin nicht in Präsenzform angeboten werden können. Zutrittszeiträume sind festzulegen; die Pausen sollten umschichtig erfolgen. Diese Umsetzungen, die erhebliche Auswirkungen auf den Lehrbetrieb haben, müssen von der Hochschulleitung vorgegeben werden.

Die Hochschulleitung muss zudem Regelungen für den Zugang und das Verlassen der Räume treffen, um das Abstandhalten von Einzelpersonen zu ermöglichen. Auch Gänge zum Händewaschen, zu Toiletten und evtl. Pausen können nur mit Abstand erfolgen. Zusätzliche Mund-Nasen-Bedeckungen sind zu erwägen, wenn der Abstand nicht zuverlässig eingehalten werden kann.

### Unterricht mit engem Kontakt unter den Studierenden 

Um in medizinischen Untersuchungskursen ohne Patient*innen, Kursen in Schauspiel und Tanz oder in Sportkursen gegenseitige Infektionen zu vermeiden, müssen alle Beteiligten eine Mund-Nasen-Bedeckung tragen. Bei Unterrichtsformen, in denen Masken den Unterrichtserfolg behindern würden (z. B. Schauspiel, Sportkurse), muss der Verzicht auf Atemschutz erwogen werden. Bei einem Verzicht auf Atemschutz sollten kleine Gruppen (ca. max. 6 Personen) zusammenarbeiten; die Personen sollten zwischen den Teams nicht wechseln.

### Unterricht mit Kontakt zu anderen Personen oder Berufsgruppen, die nicht der Universität angehören

Lehrende und Studierende sollten den Abstand von >1,5 m konsequent beibehalten, sogar möglichst eher vergrößern. Wir empfehlen das Tragen von Mund-Nasen-Schutz. Auch bei Einbeziehung von Schauspielpatient*innen, z. B. in der Vermittlung von Kommunikationstechniken, soll der Abstand bei den sprechenden Personen so groß wie möglich sein, ein Sprechabstand von 2–3 m wird von uns empfohlen. Sollte die Abstandsregel nicht zuverlässig und durchgängig umgesetzt werden können (jedoch keine engen Körperkontakte erwartet werden), empfehlen wir auch hier eine Mund-Nasen-Bedeckung für alle Beteiligten (Schauspieler*innen, Studierende und Lehrende).

### Unterricht mit engem Kontakt zu Personen, die nicht der Universität angehören und/oder als besonders gefährdet für einen schweren Verlauf einer COVID-19-Infektion eingestuft werden

Diese Unterrichtsform muss wegen des besonderen Risikos für die beteiligten Drittpersonen, i. d. R. Patient*innen in den medizinischen Studiengängen, minimiert werden und kann evtl. in der aktuellen Situation auch gar nicht zugelassen werden. Insbesondere Patient*innen mit dem Risiko, im Falle einer Infektion einen schweren Covid-19-Krankheitsverlauf zu erleiden, sind zu schützen. Die Einschätzung richtet sich nach den aktuellen Erkrankungszahlen und den Einschränkungen für andere Besucher und Angehörige. Diese Einschätzung obliegt den behandelnden Ärzt*innen. Der Besuch von Angehörigen stationärer Patient*innen hat i. d. R. Vorrang vor der studentischen Lehre. Sollte Unterricht mit Patient*innen erfolgen müssen, die ein hohes Risiko für einen schweren Verlauf haben, sind den Patient*innen zum Eigenschutz FFP2-Masken anzubieten. Alle anderen Patient*innen sollen medizinischen Mund-Nasen-Schutz (MNS) tragen [6]; die geltenden Hygieneregeln des Universitäts- oder Lehrkrankenhauses sind zu beachten. Für die Studierenden sind die konsequente Händereinigung und -desinfektion sowie das Tragen von MNS und regelmäßig gereinigter Schutzkleidung obligat. Ob das korrekte Tragen des medizinischen MNS hier nicht nur dem Fremdschutz dient, sondern auch den Träger vor der Aufnahme von Tröpfchen oder Spritzern über Mund oder Nase, z. B. aus dem Nasen-Rachen-Raum des Gegenübers schützt (Eigenschutz; [6–8]), oder ob lediglich partikelfiltrierende Halbmasken (FFP-Masken) ausreichenden Eigenschutz bieten [9, 10], ist weiterhin in Diskussion. Einfache Stoffmasken bieten jedoch einen geringeren Eigenschutz als MNS und sind hier nicht zu empfehlen [6]. Unterricht mit infektiösen, an COVID-19 erkrankten Patient*innen ist nicht möglich.

Zahnmedizinischer Unterricht am Patienten erfordert einen höheren Schutz auf Seite der Studierenden, da die Patient*innen selbstverständlich keine Mund-Bedeckung tragen können. Außerdem ist eine vermehrte Aerosolbildung bei zahnärztlichen Behandlungen möglich. Dies erfordert prinzipiell den Einsatz entsprechender Absaugtechnik [10]. Die Studierenden sollen Schutzkleidung und FFP2-Masken tragen, ergänzt durch einen Gesichtsschild, der zusätzlich vor Spritzern schützt, die mit Patient*innen-Sekreten vermischt sein könnten. Diese doppelte Schutzmaßnahme (Atemschutzmaske plus Gesichtsschild) soll auch einen zusätzlichen Schutz der Patient*innen bei Unterschreitung des Abstandes von 1,5 m im Sinne des Fremdschutzes bewirken. Infektiöse oder entsprechend verdächtige Personen dürfen von den Studierenden nicht behandelt werden.

Auch bei diesen Unterrichtsformen sind die Abstandsregeln zwischen den direkt beteiligten Personen und den momentan nicht direkt Beteiligten möglichst einzuhalten. Eine Unterschreitung der Distanz von 1,5 m zu den Risikopersonen und Patient*innen sollte nur möglichst kurzzeitig und von nur wenigen Personen erfolgen.

### Prüfungen

Wenn Präsenzprüfungen aufgrund der Prüfungsordnung nicht durch Online-Formate oder analoge Prüfungsformate (z. B. Hausarbeiten) ersetzt werden können, so ist hier im Wesentlichen sicherzustellen, dass die Abstandsregeln eingehalten werden. Für schriftliche Prüfungen sind große Räumlichkeiten, z. B. Hörsäle, notwendig. Damit die einzelnen Reihen im Hörsaal nicht zu voll besetzt werden und im Falle eines Toilettengangs nicht zu viele Menschen in der Reihe aufstehen müssen (ein aneinander Vorbeischieben ist aufgrund der Abstandsregel nicht möglich), sollte nur ca. jeder 4. oder 6. Platz besetzt werden. Hierdurch kann ein Abstand zu der schräg davor bzw. schräg dahinter sitzenden Person von ca. 1,5–2 m eingehalten werden; Abb. 1. Beim Einlass, Toilettengang und Verlassen des Prüfungsraums ist die Einhaltung der Abstandsregel und Kennzeichnung der Verkehrswege (s. oben) sicherzustellen. Aufgrund der hohen Personenzahl mit möglicherweise kurzzeitigen Unterschreitungen der Abstandsregel sollten beim Ein- und Austreten Mund-Nasen-Bedeckungen von Prüflingen und Aufsichtspersonal getragen werden.

Prüfungen, die ein Unterschreiten des Abstandes von 1,5 m erfordern, sind mit den Schutzmaßnahmen wie die entsprechenden Unterrichtseinheiten zu gestalten (s. oben).

### Exkursionen

An vielen Universitäten gilt aktuell noch ein generelles Dienstreiseverbot, welches auch die Lehrtätigkeit umfasst. Nach voraussichtlicher Lockerung dieser Regelungen sind die Vorgaben des Reiseverkehrs (Bahn, Flug) und die der zu besuchenden Institution zu beachten. Sollen wissenschaftliche Exkursionen außerhalb der Bundesrepublik Deutschland stattfinden, sind die Reisewarnungen des Auswärtigen Amtes sowie die nationalen Bestimmungen des Ziellandes zu beachten [11].

## Umsetzung

Die Vermittlung der genannten Maßnahmen kann über öffentliche Empfehlungen, Publikationen oder Leitlinien erfolgen. Die Umsetzung dieser Maßnahmen des Arbeitsschutzes in der Universität oder Hochschule ist durch die entsprechende Hochschulleitung, Präsidium bzw. die Geschäftsführung sicherzustellen. Diese sollten die Beratung durch die Sicherheitsfachkraft, die Hygienefachkraft und die Betriebsärzt*innen in Anspruch nehmen. Entsprechende Unterweisungen der Mitarbeiter*innen sind zu empfehlen. Die den Maßnahmen zugrunde zu legende Gefährdungsbeurteilung (nach ArbSchG [12]) kann mit Hilfe der Muster-Gefährdungsbeurteilung für den Interimsbetrieb der Hochschulen in der Corona-Pandemie [13] erstellt werden.

Auch die psychische Belastung der Beschäftigten und Studierenden ist in Bezug auf die veränderten Umstände zu berücksichtigen [14, 15]. Die Kommunikation der Hochschule bezüglich der betrieblichen Maßnahmen sollte klar und gut verständlich sein. Wichtige Informationen sollten allen Personen, die von den Änderungen betroffen sind, zugänglich sein (Aushänge, E‑Mail-Verteiler etc.). Wichtige Ansprechpartner sollten benannt und die Kontaktdetails ebenfalls gut zugänglich sein (z. B. online). Wir empfehlen, bei Bedarf Schulungen z. B. zu neuen digitalisierten Arbeitsformen, Hygienemaßnahmen, psychischer Gesundheit und der richtigen Verwendung von Schutzkleidung durchzuführen (Merkzettel, Videoschulungen, E‑Learning-Angebote, telefonische Beratungen bei individuellen Fragen).

Bei der Festlegung und Umsetzung der Maßnahmen sollten auch die Studierenden- und Arbeitnehmervertretungen hinzugezogen und ihre Umsetzungsideen, Sorgen und Fragen berücksichtigt werden [16].

Zusätzlich sollte das individuelle Risiko der Beschäftigten (Lehrenden und Studierenden) für einen schweren Verlauf einer SARS-CoV-2-Erkrankung im Rahmen einer arbeitsmedizinischen Beratung erwogen werden. Sowohl die lehrenden Beschäftigten als auch die Studierenden genießen im Falle einer Infektion den gesetzlichen Versicherungsschutz der Unfallkassen der Länder. Die Betriebsmediziner*innen können eine Beratung für Zugehörige von Risikogruppen oder im Erkrankungsfall auch telefonisch anbieten. Die Zugehörigkeit zu einer Risikogruppe kann dazu führen, dass eine Umsetzung an einen Arbeitsplatz oder die Bereitstellung von individuellen Lehrformen mit geringerem Risiko für eine Ansteckung (Home Office, E‑Learning und Teaching) erforderlich wird. Studierende mit erhöhtem Risiko eines schweren COVID-19-Krankheitsverlaufes sollen durch die eingeschränkte Teilnahme an Lehrveranstaltungen mit notwendiger Präsenz keinen Nachteil in ihrem Studium erfahren.

Weitere Informationen zu Risikogruppen finden sich auf der Homepage des Robert-Koch-Instituts [17] sowie in den Policy-Briefen „Müssen ältere Beschäftigte dem Arbeitsplatz fernbleiben?“ von Seidler und Petereit-Haack [18] sowie „Beschäftigte mit erhöhtem Krankheitsrisiko“ von Angerer, Kaifie-Pechmann und Tautz [19].

## Fazit und Empfehlungen

Im Hinblick auf eine weitere Öffnung des Hochschulbetriebes in Bezug auf Präsenzzeiten und praktische Erfahrungen müssen präventive Maßnahmen etabliert werden, um eine Verbreitung der COVID-19-Infektion innerhalb der Lehrbetriebe zu verlangsamen und Risikogruppen zu schützen. Eine Vielzahl an Maßnahmen der Risikominderung und des Infektionsschutzes im Rahmen des Arbeitsschutzes sind umsetzbar. Die Maßnahmen können stufenweise entsprechend des STOP-Prinzips aufgebaut werden und sich an den folgenden Prinzipien orientieren: konsequente Hygienemaßnahmen, Vermeidung nicht notwendiger Kontakte, Kontaktreduzierung und Reduzierung der Anzahl eventueller Quarantänefälle, Einhalten der Abstandsregelung und der Situation angepasstes Tragen von persönlicher Schutzausrüstung.

## Footnotes

1 MINT-Fächer: Mathematik, Informatik, Naturwissenschaft und Technik.

2 STOP = *S*ubstitution, *T*echnische, *O*rganisatorische, *P*ersönliche (Schutzmaßnahmen).
